# Supplementary material for: Clinical Significance of a Pain Scoring System for Deep Endometriosis by Pelvic Examination: Pain Score
Source: Diagnostics (Basel). 2023 May 17;13(10):1774. doi: 10.3390/diagnostics13101774 (PMC10217065; doi:10.3390/diagnostics13101774)
Supplement: Supplementary file 1 [file diagnostics-13-01774-s001.zip › Diagnostics supplementary Figure.ver.2.pptx]

## Slide 1
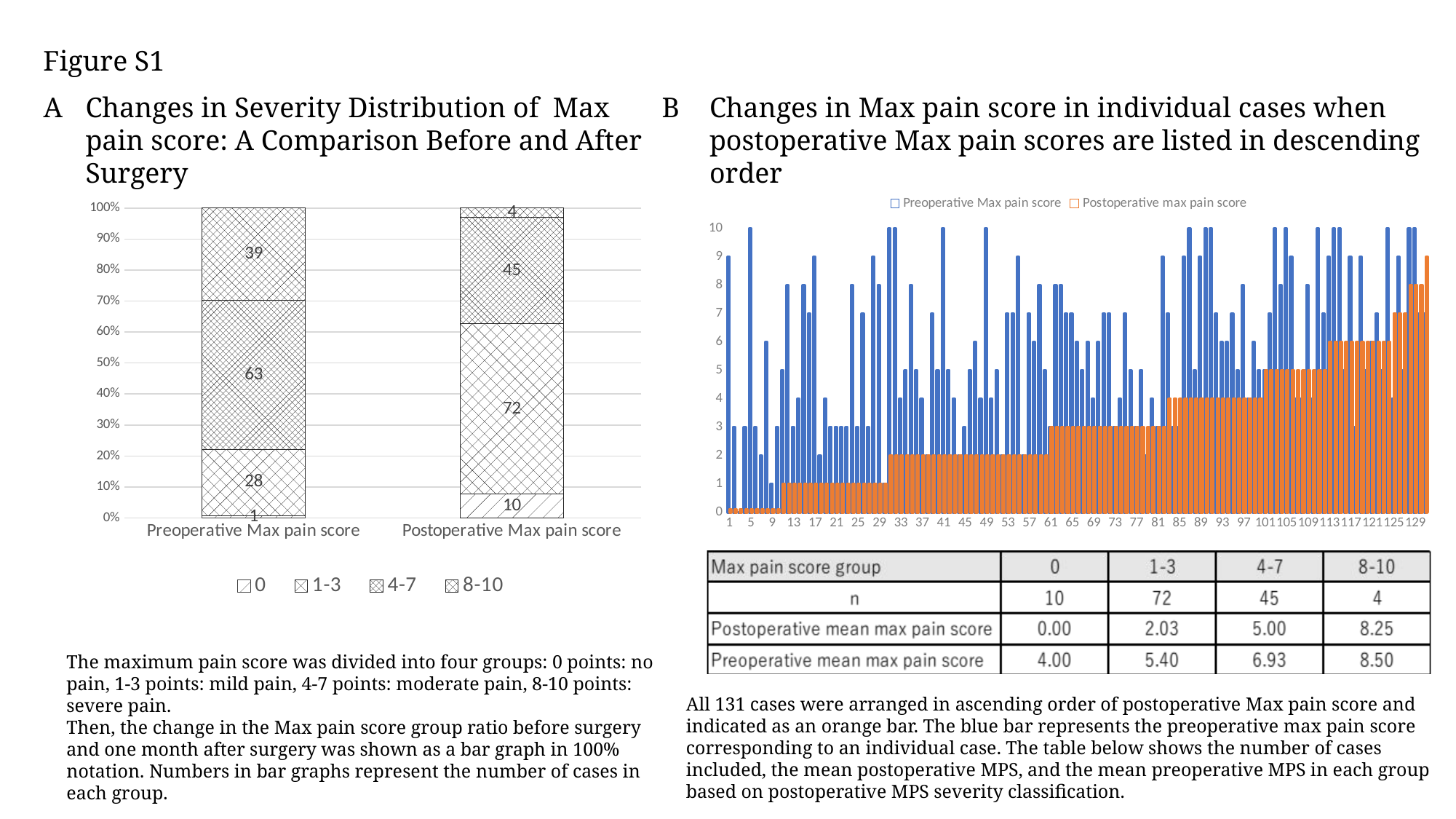

Figure S1
A
Changes in Severity Distribution of Max pain score: A Comparison Before and After Surgery
B
Changes in Max pain score in individual cases when postoperative Max pain scores are listed in descending order
### Chart
| Category | Preoperative Max pain score | Postoperative max pain score |
|---|---|---|
### Chart
| Category | 0 | 1-3 | 4-7 | 8-10 |
|---|---|---|---|---|
| Preoperative Max pain score | 1.0 | 28.0 | 63.0 | 39.0 |
| Postoperative Max pain score | 10.0 | 72.0 | 45.0 | 4.0 |The maximum pain score was divided into four groups: 0 points: no pain, 1-3 points: mild pain, 4-7 points: moderate pain, 8-10 points: severe pain.
Then, the change in the Max pain score group ratio before surgery and one month after surgery was shown as a bar graph in 100% notation. Numbers in bar graphs represent the number of cases in each group.
All 131 cases were arranged in ascending order of postoperative Max pain score and indicated as an orange bar. The blue bar represents the preoperative max pain score corresponding to an individual case. The table below shows the number of cases included, the mean postoperative MPS, and the mean preoperative MPS in each group based on postoperative MPS severity classification.

## Slide 2
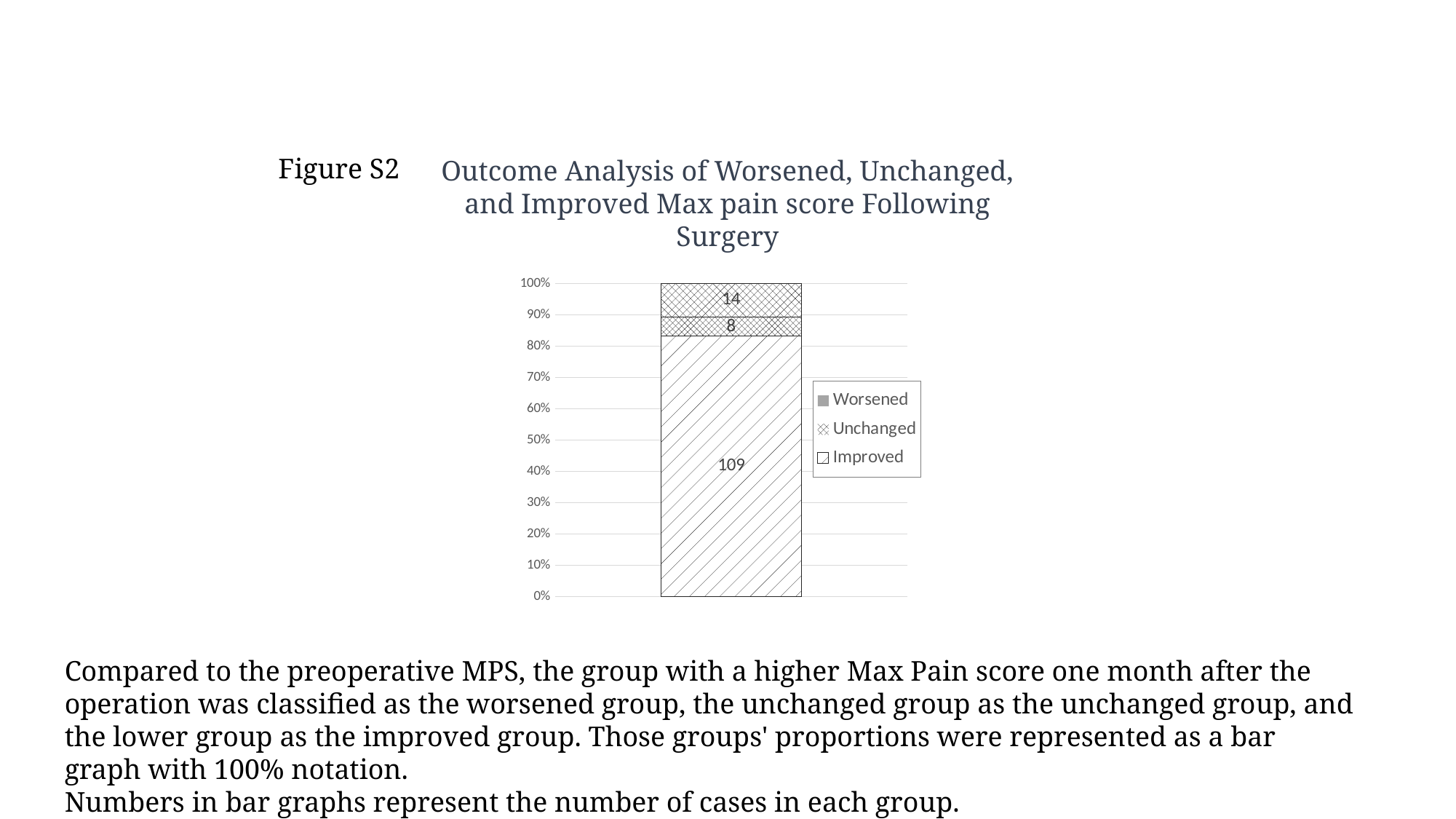

Figure S2
Outcome Analysis of Worsened, Unchanged, and Improved Max pain score Following Surgery
### Chart
| Category | Improved | Unchanged | Worsened |
|---|---|---|---|
| Postoperative improvement of Max pain score | 109.0 | 8.0 | 14.0 |Compared to the preoperative MPS, the group with a higher Max Pain score one month after the operation was classified as the worsened group, the unchanged group as the unchanged group, and the lower group as the improved group. Those groups' proportions were represented as a bar graph with 100% notation.
Numbers in bar graphs represent the number of cases in each group.
